# Molecular Hydrogen Improves Blueberry Main Fruit Traits via Metabolic Reprogramming

**DOI:** 10.3390/plants14142137

**Published:** 2025-07-10

**Authors:** Longna Li, Jiaxin Gong, Ke Jiang, Liqin Huang, Lijun Gan, Yan Zeng, Xu Cheng, Didier Pathier, Wenbiao Shen

**Affiliations:** 1 Laboratory Center of Life Sciences, College of Life Sciences, Nanjing Agricultural University, Nanjing 210095, China; lln2013034@njau.edu.cn (L.L.); 2024216040@stu.njau.edu.cn (J.G.); 2020816131@stu.njau.edu.cn (K.J.); ganlj@njau.edu.cn (L.G.); 2 College of Sciences, Nanjing Agricultural University, Nanjing 210095, China; lqhuangs@njau.edu.cn; 3 Air Liquide (China) R&D Co., Ltd., Shanghai 201108, China; yan.zeng@airliquide.com (Y.Z.); steven.cheng@airliquide.com (X.C.); didier.pathier@airliquide.com (D.P.)

**Keywords:** blueberry, fruit size, hydrogen nanobubble water, metabolism, proteome, quality

## Abstract

Fruit yield and quality improvement are challenges for researchers and farmers. This study reveals that the main fruit traits of blueberry (*Vaccinium ashei* ‘Bluegem’) were significantly improved after hydrogen (H_2_)-based irrigation, assessed by the increased single fruit weight (14.59 ± 6.66%) and fruit equatorial diameter (4.19 ± 2.39%), decreased titratable acidity, increased solid–acid and sugar–acid ratios. The enhancement of fruit quality was confirmed by the increased total volatiles, vitamin C contents, and antioxidant capacity. Using weighted protein co-expression network analysis (WPCNA), proteomic interrogation revealed that serine carboxypeptidase-like proteins I/II (SCPLI/II), ADP ribosylation factor 1/2 (ARF1/2), and UDP-glucosyltransferase 85A (UGT85A) might be functionally associated with the increased fruit weight and size driven by H_2_. Reduced organic acid accumulation was caused by the regulation of the specific enzymes involved in sucrose metabolism (e.g., α-amylase, endoglucanase, β-glucosidase, etc.). H_2_ regulation of fatty acid degradation (e.g., acyl CoA oxidase 1 (ACX1), acetyl CoA acyltransferase 1 (ACAA1), etc.) and phenylpropanoid metabolism were used to explain the improved fruit aroma and anthocyanin accumulation. Meanwhile, the upregulated heat shock protein 20/70 matched with the enhanced antioxidant activity. Together, this study provides a novel approach for yield and quality improvement in horticultural crops.

## 1. Introduction

Blueberry (*Vaccinium* spp.) is rich in bioactive compounds such as anthocyanins, flavonoids, and other polyphenolic compounds and is considered to be one of the most nutritious and healthy foods [1]. By 2020, China had become the leading country globally in terms of both blueberry cultivation area and total production, encompassing fresh and processed fruit outputs [2]. As of 2024, the cultivation area expanded to 95,880 hm^2^, with total output reaching 780,000 t. However, the average yield per unit area and overall fruit quality remain suboptimal. Major blueberry-producing regions in China include Yunnan, Guizhou, Liaoning, Sichuan, Shandong, and Jiangsu provinces. Among various cultivars, rabbiteye blueberries (*Vaccinium ashei* Reade) have good resistance to damp heat and have a large planting area in the Yangtze River Basin [3]. The sustained growth in consumer demand for both fresh consumption and processing applications continues to drive agricultural producers and researchers to focus on enhancing yield and improving fruit quality.

Hydrogen (H_2_) is widely used as an industrial and medical gas [4,5]. Meanwhile, the production and release of H_2_ have been observed in algae [6], animals [7], and plants [8]. Conventionally, H_2_ production in animals results from gut bacterial fermentation [7]. In plants, although there have been no algal hydrogenase-like genes or proteins, H_2_ can be produced under normal conditions [8] or as a result of various stress conditions and phytohormone induction [9,10]. Several laboratories discovered that both the increased endogenous H_2_ level and exogenous H_2_ supplementation not only induced root formation [11] acting as a bioactive molecule, but also enhanced plant tolerance against drought [12], salt [9], cold [13], and heavy metal exposure [14]. Accordingly, its potential application in agriculture has attracted substantial attention [15]. Since H_2_ has diffusivity and potential flammability (range 4% and 75% *v*/*v* in air), hydrogen nanobubble water (HNW) is currently used for H_2_ supplementation in both laboratory and field trials [16]. When dissolved H_2_ concentration is <0.5 mM (0.001‰, *v*/*v*), HNW has phytological activity. It was further confirmed that it improved agronomic traits, including higher yield and quality, in field-grown rice [17], Chinese cabbage [18], strawberry [19], and tomato [20] after being irrigated with HNW during growth seasons.

Accumulating studies have indicated that serine carboxypeptidase-like (SCPL) proteins participate in various biological processes, including control of cell division [21], production of secondary metabolites [22], and mobilization of storage proteins [23]. For example, *grain size 5* (*GS5*) encodes a serine carboxypeptidase-like (SCPL) group II protein and was found to positively regulate grain size and weight in rice [21] and wheat [24]. Previous studies observed that HNW increases the length, width, and thickness of brown/rough rice and white rice through modulating the yield-related genes, including *GS3* and *GS5* [17]. Moreover, ADP ribosylation factor (ARF) family proteins regulate membrane traffic and modulate organelle structure in eukaryotic cells through a regulated cycle of GTP binding and hydrolysis [25]. Studies discovered that overexpression of maize *ZmARF1* and *ZmARF2* in *Arabidopsis* could promote plant growth by increasing cell size, resulting in increased plant height, enlarged leaves, and seed size [26,27]. Mover, UDP-glucosyltransferase (UGTs) glycosylate a variety of specialized metabolites, such as flavonoids, phenols, terpenoids, anthocyanins, and plant hormones, modifying secondary metabolites and affecting plant growth and development [28]. Studies have found that seven genes in the AtUGT85A subfamily (*AtUGT85A1*, *2*, *3*, *4*, *5*, and *7*) are highly expressed in actively dividing tissues of *Arabidopsis*, and silencing of *AtUGT85A7* impaired auxin responsiveness, thus inhibiting root and leaf growth and development [29]. Currently, the mechanism underlying the regulation of HNW on seed or fruit development, as well as the potential targets, remains unclear.

Additionally, HNW was observed to regulate plant secondary metabolism. It reduced titrable acid content and increased sugar content in both strawberry [19] and tomato [20], leading to a significant increase in the sugar–acid ratio. However, the mechanism of H_2_-regulated organic acid metabolism has not been fully elucidated to date. Moreover, HNW altered the profile of strawberry volatile compounds, assessed by the stimulated gene expression related to ester and furanone derivatives synthesis, as well as linalool and nerolidol synthesis [19]. Increased anthocyanin accumulation was also observed in H_2_-treated radish sprouts [30]. These changes were attributed to the effects of H_2_ on phenylpropanoid metabolism modulation. Transcriptomic analysis showed that under UV-A radiation, H_2_ upregulated genes involved in the anthocyanin biosynthesis in the hypocotyls of radish seedlings, including *phenylalanine ammonialyase* (*PAL*), *chalcone synthase* (*CHS*), *dihydroflavonol reductase* (*DFR*), etc. [31]. Further metabolomics and transcriptomic analyses showed that the main active components of *Ficus hirta* Vahl were improved by H_2_ through regulating phenylpropanoid biosynthesis and metabolism [32]. Since the metabolism regulation achieved by HNW might be modulated at both transcriptional and protein levels, the related mechanism needs further elucidation, especially at the protein level.

Previous studies showed that plants of different species or different growth stages, such as irrigated conventional rice, transgenic rice, and wild rice species [33], as well as cucumber seedlings and rose flowers [34], have different responses to H_2_. Different from annual crops studied previously, blueberries are woody. However, studies on the effects of H_2_ on woody plants are limited, and there are only *F. hirta* roots [32] and cut rose flowers [35]. Here, we aim to investigate whether or how HNW irrigation influences blueberry yield and quality, especially using proteomic technology combined with biochemical and molecular biology approaches to provide insight into the possible molecular mechanisms from the perspective of metabolic regulation. This study might provide a reference for the improvement in yield and quality in horticultural crops and enrich the understanding of the metabolic regulatory effects of H_2_.

## 2. Results

### 2.1. H_2_-Based Irrigation Improves Blueberry Fruit Size and Weight

**Figure 1:**
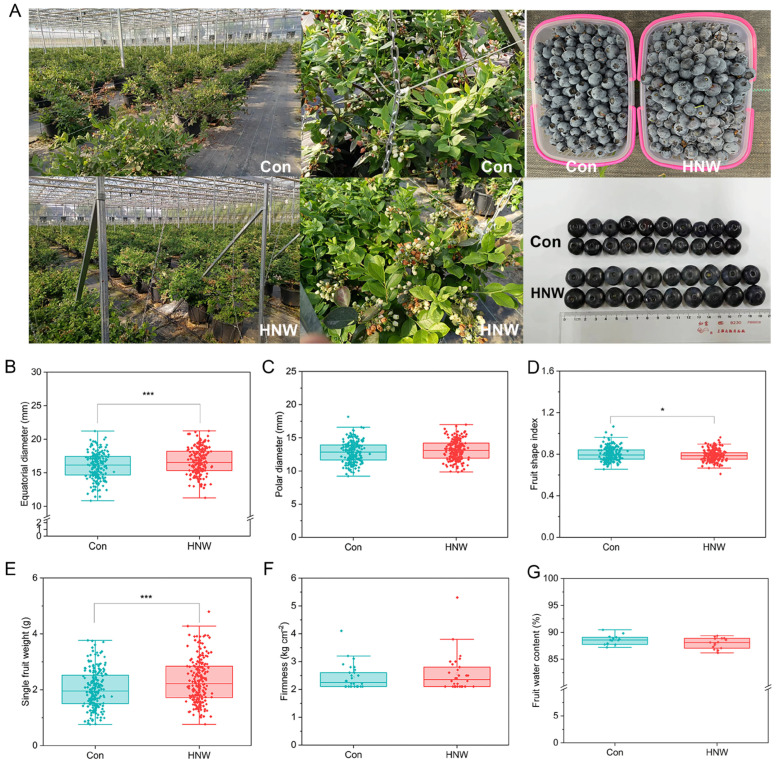
Growth and fruit of blueberry after hydrogen nanobubble water (HNW) irrigation. Photos of blueberry plants, flowers, and fruits were taken on 7 May 2022 and 15 June 2022, respectively (**A**). Changes in fruit equatorial diameter (**B**), polar diameter (**C**), fruit shape index (**D**), single-fruit weight (**E**), firmness (**F**), and fruit water content (**G**) in response to HNW. The number of samples in (**B**–**E**), (**F**), and (**G**) is 198 fruits (66 × 3), 30 fruits (10 × 3), and 15 fruits (5 × 3), respectively. * and *** indicate *p* < 0.05 and 0.001, respectively (*t*-test).

As expected, HNW irrigation improved the growth performance of blueberry plants and fruit size (Figure 1A). Unlike the changes in polar diameter, the fruit equatorial diameter was increased by 4.19 ± 2.39% (*p* < 0.001) in comparison with the control group, leading to a slight but significant decrease in the fruit shape index (*p* < 0.05) (Figure 1B–D). The single fruit weight of blueberries irrigated with HNW was 2.32 ± 0.78 g, also showing a significant increase (14.59 ± 6.66%; *p* < 0.001; Figure 1E). However, there were no obvious effects on the firmness and water content of blueberry fruits (Figure 1F,G).

### 2.2. Effects of H_2_-Based Irrigation on Blueberry Flavor Quality

Compared to the control group, although HNW irrigation did not significantly influence soluble solids content (SSC) and total soluble sugars (TSS) in blueberries, it strikingly reduced titratable acidity (TA; −22.63 ± 9.67%; *p* < 0.05), consequently leading to a significant increase in SSC/TA (41.17 ± 9.63%; *p* < 0.01) and TSS/TA (33.93 ± 8.18%; *p* < 0.05) (Table 1).

**Figure 2:**
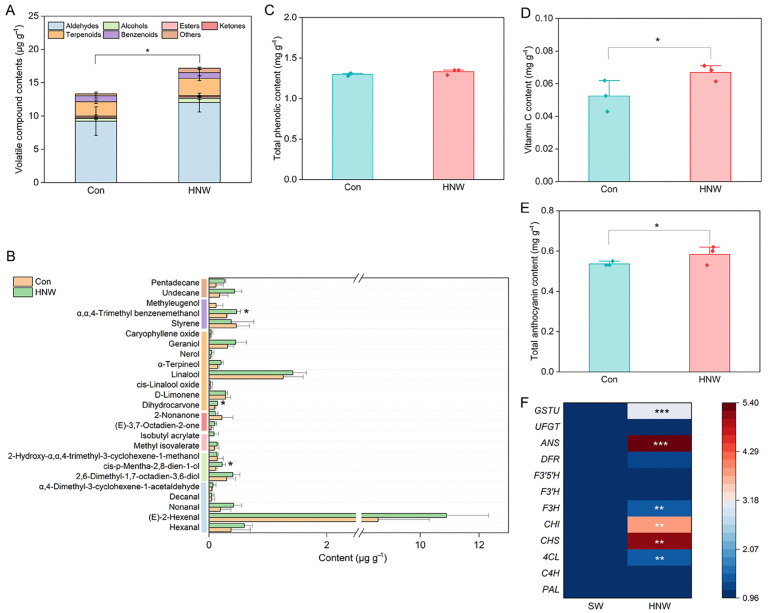
Effects of hydrogen nanobubble water (HNW) on contents of total volatiles (**A**), representative volatiles (**B**), total phenols (**C**), vitamin C (**D**), total anthocyanins (**E**), and anthocyanin biosynthesis-related genes expression profiles (**F**) in blueberry fruits. Volatile concentration is expressed as μg g^−1^ of fresh weight, equivalent to ethyl decanoate. Values are expressed as mean ± SD (three replicates and ten fruits per each). * *p* < 0.05, ** *p* < 0.01, and *** *p* < 0.001 (*t*-test). 4CL: 4-coumarate:CoA ligase; ANS: anthocyanidin synthase; C4H: cinnamic acid-4-hydroxylase; CHI: chalcone isomerase; CHS: chalcone synthase; DFR: dihydroflavonol 4-reductase; F3H: flavanone-3-hydroxylase; F3′H: flavonoid 3′-hydroxylase; F3′5′H: flavonoid 3′,5′-hydroxylase; GSTU: tau class glutathione S-transferase; PAL: phenylalanine ammonia lyase; UFGT: UDP-glycose flavonoid glycosyltransferase.

A total of 25 major volatile compounds were identified in blueberry fruits, including aldehydes, alcohols, esters, ketones, terpenoids, benzenoids, and others. HNW had no effect on the types of volatile compounds, but altered their contents (Figure 2A). Except for ketones, the contents of other types, especially terpenoids (20.12 ± 8.22%), were increased by HNW to varying degrees, resulting in a significant increase in total volatile content (30.67 ± 17.13%; *p* < 0.05). Among these, HNW increased the contents of α,α,4-trimethyl benzenemethanol, dihydrocarvone, and cis-p-mentha-2,8-dien-1-ol (*p* < 0.05; Figure 2B).

### 2.3. Antioxidant Capacity of Blueberries Is Enhanced by H_2_-Based Irrigation

Although HNW had no significant effect on the content of total phenolic (TP; Figure 2C), it remarkably increased vitamin C (VC) and total anthocyanin content by 29.87 ± 17.29% and 7.92 ± 1.64%, respectively (*p* < 0.05; Figure 2D,E). Further gene expression analysis showed that certain anthocyanin biosynthesis-related genes, including *4-coumarate:CoA ligase* (*4CL*), *CHS*, *chalcone isomerase* (*CHI*), *flavanone-3-hydroxylase* (*F3H*), *anthocyanidin synthase* (*ANS*), and *tau class glutathione S-transferase* (*GSTU*) in fruits were significantly upregulated by HNW irrigation (*p* < 0.01 or 0.001; Figure 2F).

**Figure 3:**
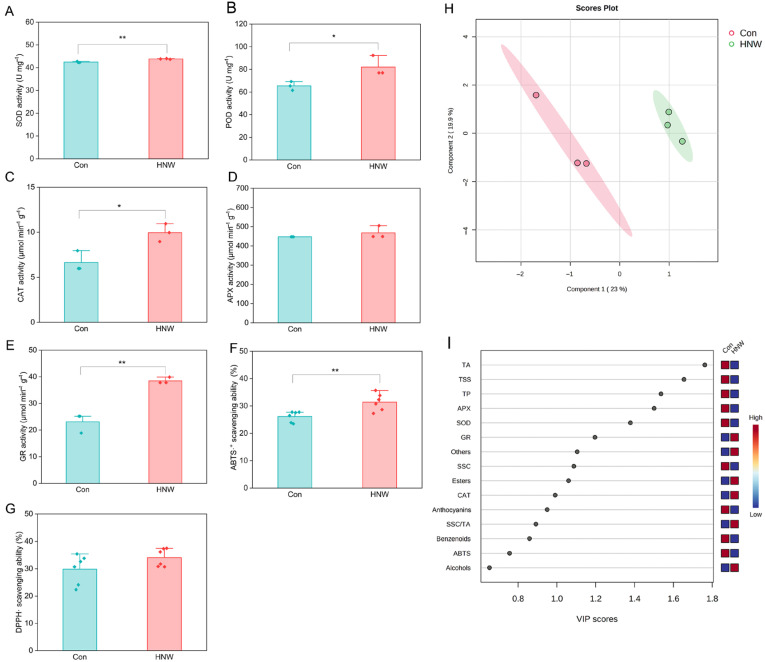
Effects of hydrogen nanobubble water (HNW) on antioxidant capacity and multivariate analysis of quality-related characters in blueberry fruits. The activities of superoxide dismutase (SOD; (**A**)), peroxidase (POD; (**B**)), catalase (CAT; (**C**)), ascorbate peroxidase (APX; (**D**)), and glutathione reductase (GR; (**E**)). The scavenging abilities of 2,2′-azino-bis(3-ethylbenzothiazoline-6-sulfonic acid) radical (ABTS·^+^; (**F**)) and 1,1-diphenyl-2-picryl-hydrazyl radical (DPPH·; (**G**)). Values are expressed as mean ± SD (three replicates and ten fruits per each). * *p* < 0.05 and ** *p* < 0.01 (*t*-test). Score plot of partial least squares-discriminant analysis (PLS-DA; (**H**)). Variable importance in projection (VIP; (**I**)) scores listed importance of quality-related characters. The colored boxes on the right indicate the relative abundances of the corresponding characters.

It was clearly observed that HNW increased the activities of antioxidant enzymes, including superoxide dismutase (SOD), peroxidase (POD), catalase (CAT), and glutathione reductase (GR) (*p* < 0.05 or 0.01), with the exception of ascorbate peroxidase (APX) (Figure 3A–E). Consistently, the antioxidant capacity assessed by 2,2′-azino-bis(3-ethylbenzothiazoline-6-sulfonic acid) radical (ABTS·^+^) scavenging ability was enhanced after HNW irrigation (*p* < 0.01; Figure 3F), while there was no significant change in 1,1-diphenyl-2-picryl-hydrazyl radical (DPPH·) scavenging abilities (Figure 3G).

### 2.4. PLS-DA Analysis of Quality Characteristics in Blueberry Fruits After H_2_-Based Irrigation

PLS-DA analysis was applied to differentiate the HNW group and the control group (Figure 3H). Two components explained 23% (component 1) and 19.9% (component 2) of the total variance. Component 1 clearly separated the HNW treatment from the control group. Further Variable Importance in Projection (VIP) analysis of PLS-DA identified the most important characters involved in discrimination between the HNW group and the control group (VIP score > 1.0, Figure 3I). The characters were associated with flavor quality, including TA, TSS, other volatile compounds, SSC, and esters contents, as well as characters associated with antioxidant properties, including TP, APX, SOD, GR, and CAT activities.

### 2.5. Protein Changes in Response to H_2_-Based Irrigation

Among the 4756 proteins identified (Supplementary Table S1), 59 and 83 proteins were found to be significantly upregulated and downregulated (control vs. HNW; Supplementary Table S2). The main Kyoto Encyclopedia of Genes and Genomes (KEGG) functional classifications of these differentially expressed proteins (DEPs) revealed that metabolic pathways (51 proteins), biosynthesis of secondary metabolites (32 proteins), starch and sucrose metabolism (eight proteins), glycerophospholipid metabolism (six proteins), fatty acid degradation (five proteins), and amino sugar and nucleotide sugar metabolism (five proteins) were significantly enriched (Supplementary Figure S1). These results indicated that changes in the above major metabolic pathways might contribute to HNW-induced improvement in blueberry fruit weight, size, and quality.

### 2.6. Identification of Proteins That Correlated Most with Main Fruit Traits

**Figure 4:**
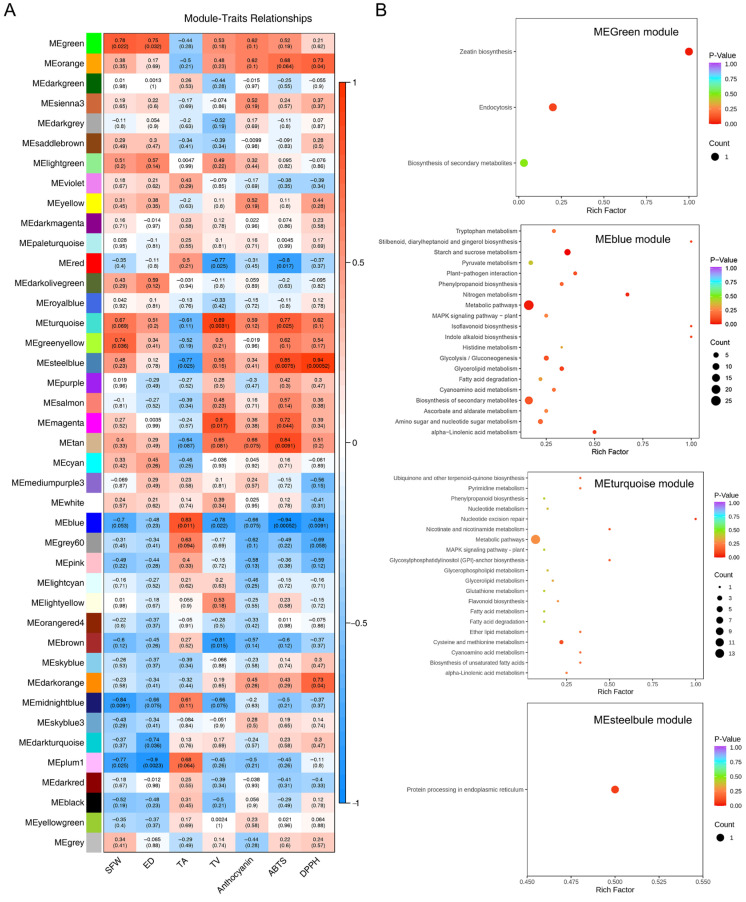
The proteomic response of blueberry fruits after hydrogen nanobubble water (HNW) irrigation. Correlation between modules and blueberry yield and quality traits (**A**). Each row of the left heatmap represents a module, and each column represents a character of blueberry yield and quality. The value in each cell represents the Pearson correlation coefficient between the module and the trait, and the value in parentheses in each cell represents the *p*-value. Red color represents the positive correlation between module and trait, and blue color represents the negative correlation. ABTS: ABTS·^+^ scavenging ability; DPPH: DPPH· scavenging ability; SFW: single-fruit weight; ED: equatorial diameter; TA: titratable acidity; TV: total volatile content. KEGG enrichment analysis of proteins in the corresponding modules that were highly positively correlated with traits (**B**); *p*-value ranks of the 20 top.

Weighted protein co-expression network analysis (WPCNA) was performed to identify candidate proteins involved in HNW-improved blueberry fruit mass and quality. A total of 41 modules were obtained, represented by different colors based on hierarchical clustering (Supplementary Figure S2). Correlation analysis further showed a significant positive correlation between the MEgreen module and single-fruit weight (r = 0.78, *p* = 0.022) and fruit diameter (r = 0.75, *p* = 0.032), respectively (Figure 4A). The similar correlation was also observed between the MEblue module and titratable acidity (r = 0.83, *p* = 0.011), the MEturquoise module and total volatile compounds (r = 0.89, *p* = 0.0031), as well as the MEsteelblue module and ABTS (r = 0.85, *p* = 0.0075) and DPPH radical scavenging capacity (r = 0.94, *p* = 0.00052), respectively, while no module was significantly correlated with anthocyanin content.

In the MEgreen module (total 251 proteins), four upregulated DEPs were observed, including serine carboxypeptidase-like protein I/II (SCPLI/II; id278 and id1792), ADP-ribosylation factor 1 (ARF1; id3005), and UDP-glucosyltransferase 85A (UGT85A; id1810) (Table 2). Among these, UGT85A was involved in the enrichment pathway for the biosynthesis of zeatin (*p* < 0.05; Figure 4B).

**Figure 5:**
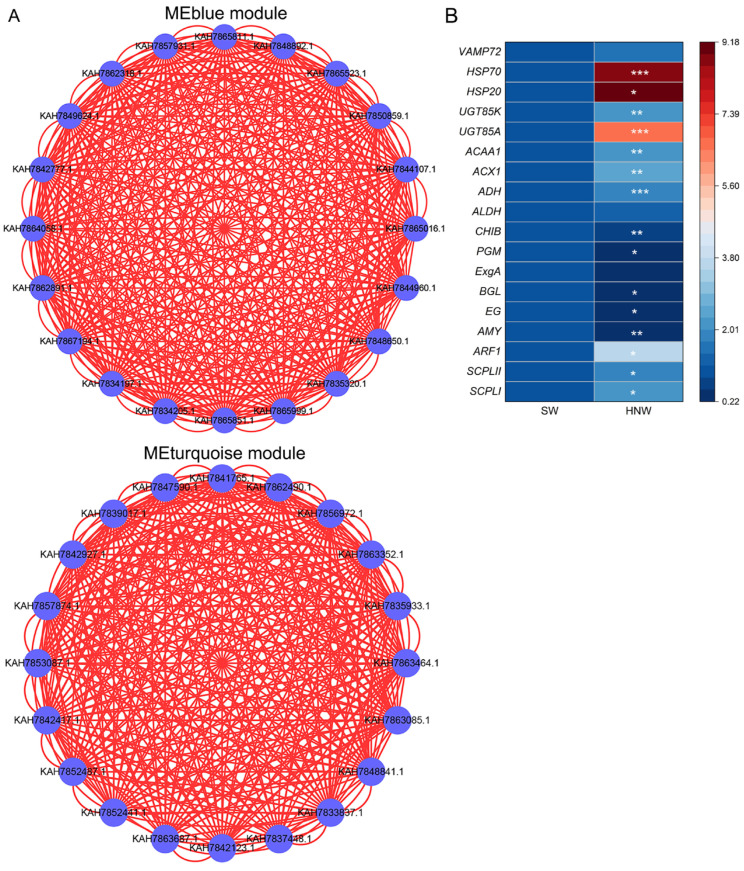
Co-expression networks of proteins in MEblue and MEturquoise modules (**A**) and the expression levels of genes coding differentially expressed proteins (**B**). Nodes represent the top 20 proteins for connectivity (K value) in each module, labeled by NCBI accession number, and lines represent the linear relationship. ACAA1: acetyl-CoA acyltransferase 1; ACX1: acyl-CoA oxidase; ADH: alcohol dehydrogenase; ALDH: alcohol dehydrogenase; AMY: α-amylase; ARF1: ADP-ribosylation factor 1; BGL: β-glucosidase; CHIB: endochitinase B; EG: endoglucanase; ExgA: glucan 1,3-β-glucosidase; HSP20/70: heat shock 20/70kDa protein; PGM: phosphoglucomutase; SCPLI/II: serine carboxypeptidase-like protein I/II; UGT85A/85K: UDP-glucosyltransferase 85A/85K; VAMP72: vesicle-associated membrane protein 72. * *p* < 0.05, ** *p* < 0.01 and *** *p* < 0.001 (*t*-test; n = 3).

There were 60 DEPs in the MEblue module (total 458 proteins), which were mainly related to carbohydrate metabolism, included starch and sucrose metabolism (eight DEPs; *p* = 0.002; e.g., α-amylase, AMY; endoglucanase, EG; β-glucosidase, BGL; etc.), glycolysis/gluconeogenesis (four DEPs; *p* = 0.122; e.g., phosphoglucomutase, PGM; aldehyde dehydrogenase, ALDH; alcohol dehydrogenase, ADH), and amino sugar and nucleotide sugar metabolism (four DEPs; *p* = 0.171; phosphoglucomutase, PGM; endochitinase B, CHIB; chitinase, Chi; Figure 4B, Table 2 and Supplementary Table S3). Moreover, the top 20 proteins were selected for visualization of the interaction network in this module, and AMY, CHIB, ALDH, and ADH were listed (Figure 5A), indicating that these proteins might be closely related to the HNW decrease in blueberry organic acid.

There were 35 DEPs within the MEturquoise module (total 562 proteins). The significantly enriched pathways in this module were unsaturated fatty acid biosynthesis and α-linolenic acid metabolism (*p* < 0.05, Figure 4B), which included two DEPs, Acyl-CoA oxidase (ACX1), and acetyl-CoA acyltransferase 1 (ACAA) (Table 2 and Supplementary Table S3). However, among the top 20 proteins in connectivity ranking in this module, there were no known DEPs directly related to volatile compound synthesis (Figure 5A). The connectivity rankings for ACX1 and ACAA1 were 97 and 167, respectively. The above DEPs might play important roles in the accumulation of volatile compounds in blueberries stimulated by HNW irrigation.

The MEsteelblue module (total 47 proteins) had two DEPs, and they were heat shock protein 20 (HSP20) and vesicle-associated membrane protein 72 (VAMP72) (Table 2 and Supplementary Table S3). Meanwhile, there were no significantly enriched metabolic pathways. Additionally, the MEturquoise module showed significant correlation with ABTS·^+^ scavenging capacity (Figure 4A), where peroxidase (POD), glutathione S-transferase (GST), HSP20, and HSP70 were identified (Table 2).

### 2.7. Validation of Protein Profiles by qPCR

In order to validate the quality of the proteomics, 19 DEPs were selected for qPCR analysis (Figure 5B). Among these, the expression patterns of genes encoding SCPLI/II, ARF1, AMY, EG, BGL, PGM, CHIB, ACX1, ACAA1, ADH, UGT85A, UGT85K, HSP20, and HSP70 were consistent with the results in TMT-labeled proteomics data (Table 2), except for genes encoding ExgA, ALDH, and VAMP72. These results suggested that the quality of our proteomics data might be acceptable.

## 3. Discussion

Consistent with the previous studies on the HNW-irrigated rice [17], Chinese cabbages [18], tomatoes [20], and strawberries [19,36], this study showed that H_2_-based irrigation not only improved blueberry fruit quality, including the levels of flavor-related and antioxidant compounds, but also the fruit mass (Figure 1, Figure 2 and Figure 3 and Table 1). These results indicated that the HNW might have great potential in horticultural crop production. Considering the limited consumption of H_2_ (1–2 USD/kg H_2_ in the United States, Europe, and China), the cost of H_2_-based irrigation primarily depends on the equipment and labor expenses [37]. Therefore, the technology of H_2_ supplementation in horticulture emerges as both economically practicable and viable.

### 3.1. The Genes/Proteins for the H_2_-Enhanced Blueberry Fruit Size and Weight

Ample studies demonstrated that SCPLs, ARFs, and UGTs are involved in multiple plant physiological processes, including regulating plant growth and development, directly or indirectly resulting in seed size and weight improvement [21,22,23]. For instance, higher expression of *extra carpels and seeds 1* (*ECS1*), *OsGS5*, and *TtGS5* (all encoding SCPLII proteins), *ZmARF1*/*2* were confirmed to control seed size or/and weight in *Arabidopsis* [26,27,38], rice [21], wheat [24], respectively. These SCPLs participate in cell division [21,24,38], and ARFs regulate cell size [26,27]. Moreover, seven AtUGT85A genes identified in *Arabidopsis* might be associated with root and leaf cell cycle regulation [29]. Previously, HNW was observed to improve rice grain size and weight, accompanied by an increase in *GS5* expression. In this study, based on the proteomic data and WPCNA analysis, two SCPL proteins, an ARF1 and a UGT85A, were closely associated with the HNW-increased blueberry fruit size and weight (Table 2), indicating that the beneficial effect of HNW might be attributed to the modulation of cell division and cell size.

### 3.2. H_2_-Reduced Organic Acids Accumulation Possibly by Regulating Sugar Metabolism

In contrast to the findings in strawberries [19] and tomatoes [20], HNW slightly increased soluble sugar content in blueberries but significantly reduced TA level (Table 1). These results indicated that H_2_ regulation of sugar–acid metabolism might appear to be species-dependent. In the tricarboxylic acid cycle (TCA), glyoxylic acid cycle, and other organic acid metabolism pathways, there were no DEPs observed. However, WPCNA analysis revealed that certain DEPs involved in starch and sucrose metabolism, amino sugar, nucleotide sugar metabolism, and glycolysis, including AMY, EG, BGL, GN1/2/3, PGM, CHIB, ADH, and ALDH, were significantly correlated with HNW-reduced organic acid content (Figure 4 and Table 2). Starchless *pgm* mutants of *Arabidopsis* showed lower levels of soluble sugars, malic acid, and citric acid compared to the wild type [39]. Additionally, ALDH and ADH, which facilitate the interconversion between alcohols to aldehydes, are involved in various metabolic pathways in higher plants, including glycolysis/gluconeogenesis, interconversion of pentose and glucuronate, fatty acid and amino acid degradation, thus not only affecting fruit organic acid metabolism but also volatile substance metabolism [40,41]. In glycolysis/gluconeogenesis, downregulation of ALDH and ADH may slow down the entry of carbon into the citrate cycle. Therefore, in this study, HNW-decreased TA might be attributable to the down-regulated expression (abundance) of proteins and their corresponding genes involved in sugar metabolism at the late stage of blueberry ripening.

### 3.3. H_2_-Based Irrigation Regulates Fatty Acid Metabolism to Influence Fruit Aroma Formation

In fruits, aroma compounds, including alcohols, aldehydes, esters, and ketones, are predominantly generated through the lipoxygenase (LOX) pathways and β-oxidation [42]. ALDH and ADH both participate in the LOX pathway. It was reported that *SlscADH1* inhibition increased the concentrations of C5 and C6 volatile compounds in fruits [43]. ACX1 and ACAA1 are the first and last enzymes that participate in fatty acid β-oxidation, respectively [44,45]. Expression of *PpACX1* and activity of ACX were positively correlated with volatile lactones in peach [46]. In this study, we noticed that HNW upregulated the expression of ACX1 and ACAA1 (Table 2). Since the stimulated ACX1 and ACAA1 promoted acetyl-CoA synthesis [47,48], we further speculated that the increased acetyl-CoA might promote terpenoids synthesis through the mevalonate (MVA) pathway [47]. This speculation was confirmed by the higher levels of terpenoids achieved by H_2_-based irrigation in blueberry fruits (Figure 2). Furthermore, two differentially expressed UGTs were identified, namely, UGT85A and UGT85K (Figure 4 and Table 2). They were found to be involved in triterpenoids accumulation in *Cyclocarya paliurus* leaves [49]. Combined, the regulation of the above protein might collectively lead to changes in the volatile profiles of HNW-treated blueberries.

### 3.4. The Reprogramming of Phenylpropanoid Metabolism by H_2_-Based Irrigation to Improve Anthocyanins Accumulation

It is well documented that reprogramming of phenylpropanoid metabolism could affect the contents of plant flavonoids, anthocyanins, and aroma, thereby affecting the quality of horticultural products [50]. For example, RNAi-mediated *FaCHS* silencing and simultaneous heterologous overexpression of an eugenol synthase gene stimulated the accumulation of coumaroyl-CoA-derived metabolites and redirected the carbon flux from the anthocyanin/flavonoid pathway to volatile phenol compound synthesis, thus affecting strawberry aroma [51]. Additionally, the expression of a transgenic anti-F3H gene in carnation flowers results in the emission of higher levels of methyl benzoate [52]. Consistent with the previous study on radish sprouts [30], HNW increased the anthocyanin content, along with the elevated expression of *4CL*, *CHS*, *CHI*, *F3H*, *ANS*, and *GSTU* transcripts in blueberry fruits (Figure 2F). Proteomic analysis further showed an increase in the abundance of CHI protein that matched with its gene expression, while the abundances of CADs required for lignin synthesis, and C12RT1 and PGT1 involved in catalyzing flavone and flavonol glycosylation were decreased (Supporting Information, Table S3). The above proteomic results were consistent with the metabolomic results of our previous study, in which an increase in phenolic acids and flavonoids accumulation of blueberry fruits was achieved by HNW irrigation [53]. These results indicated that HNW might affect the composition of flavonoid compounds in blueberry fruits through reprogramming phenylpropanoid metabolism.

### 3.5. Stress Response Proteins Involved in HNW-Enhanced Antioxidant Ability

The enzymatic components of the plant antioxidant system, including SOD, CAT, POD, GR, and GST, collectively maintain redox homeostasis. Additionally, VAMPs and HSPs mediate defense responses against abiotic stresses (heat, drought, salinity) and biotic challenges (pathogen infection) [54,55]. *Arabidopsis* VAMP721/722 (VAMP72 subfamily) regulated vesicle exocytosis and contributed to plant resistance to powdery mildew [56]. Moreover, apple *MsHsp16.9* (a member of the HSP20 family) was strongly induced by high temperature and overexpressing of *MsHsp16.9*, while in *Arabidopsis*, it showed increased heat tolerance [57]. Additionally, MsHsp16.9 might function alongside AtHSP70 to maintain protein homeostasis, enhance antioxidant enzyme activity, and alleviate oxidative damage.

In this study, as expected, HNW increased the levels of VC and anthocyanins in blueberries (Figure 2D,E), and the activities of SOD, CAT, POD, and GR (Figure 3A–C,E). Further proteomic analysis revealed an increase in POD, GST, VAMP72, HSP20, and HSP70 protein abundance (Table 2). These changes matched the enhanced antioxidant capacity, as assessed by ABTS·^+^ scavenging ability (Figure 3F) and were also supported by the results of WPCNA analysis (Figure 4 and Table 2). In our experiments, the ‘Bluegem’ blueberry matures from May to June (Shanghai, China), facing the relatively hottest environment in the greenhouse. Therefore, HNW stimulated VAMP72, HSP20, and HSP70, thus maintaining higher antioxidant enzyme activity and enhancing the fruit heat tolerance.

## 4. Materials and Methods

### 4.1. Plant Material and Experimental Design

The pot experiment was carried out in Qingpu Modern Agriculture Park, Shanghai, China (121.03° E, 30.97° N) from February to June 2022. Three-year-old blueberry plants (*Vaccinium ashei* ‘Bluegem’) were grown in the greenhouse with an automatic drip irrigation system. The plants were irrigated with a nutrient solution prepared with the hydrogen nanobubble water (HNW) and the ordinary water (control group). Control and HNW treatments were set up in three replicates, with 154 plants per replicate. The irrigation details were shown in Supplementary Table S4. Plants were cultivated according to current commercial practices for blueberry greenhouse cultivation in Shanghai. The fully matured fruits (both the exocarp and pedicel had turned blue with pedicel detachment initiation) were harvested in June 2022 for the analyses of main fruit traits.

### 4.2. The Preparation of Hydrogen Nanobubble Nutrient Solution

The hydrogen nanobubble water generation system (Liquid Air (China) R&D Co., Ltd., Shanghai, China) was integrated into the fertigation system of the blueberry plantation. The concentration of dissolved H_2_ in the hydrogen nanobubble nutrient solution was ~1.0 mg L^−1^ with >12 h residence time. The nanobubbles were ~300 nm. The composition of the nutrient solution is shown in Supplementary Table S5.

### 4.3. Measurement of Fruit Phenotypic Traits

Single-fruit weight, the horizontal and vertical diameters, and fruit shape index (horizontal diameter/vertical diameter) were measured. There were three replicates with 66 fruits per replicate performed for the treatments. The fruit hardness was measured using the GY-1 fruit hardness tester (Sanhe Measuring Instrument, Wenzhou, China). Control and HNW treatments were performed with three replicates, and ten fruits per replicate, respectively. The water content (%) was calculated as the difference between fresh and dry weights. There were three replicates with five fruits per replicate.

### 4.4. Extraction and Analyses of Flavor Characteristics

Soluble solid content (SSC) was determined by using the LB32T handheld refractometer (SWEVY, Guangzhou, China). There were three replicates with 10 fruits per replicate performed for the control and HNW treatments. Additionally, 30 fruit samples (10 × 3) were ground into a powder using a liquid nitrogen grinder (A11, IKA, Staufen, Germany), then stored at −80 °C for the following analyses. The content of total soluble sugars (TSS) and titratable acidity (TA) were determined as described previously, using anthrone colorimetric and titration methods, respectively [19]. The absorbance of the fruit sample was measured at 620 nm, and the results were calculated using a standard curve prepared with sucrose. The titration was performed using 0.1 M NaOH to provide an endpoint at pH 8.2. The volatiles of fruits were collected and analyzed following the previous method [19], using ethyl decanoate as internal standard, the SPME fiber (50/30 μm DVB/CAR/PDMS, Supelco, Bellefonte, PA, USA), and a 320-MS gas chromatograph–mass spectrometer. The identification of the volatile compounds was performed by comparing the mass spectra of the samples with the NIST11 standard library. The relative amount of a component was calculated in reference to the internal standard (μg g^−1^ fresh weight). Each sample was analyzed in triplicate.

### 4.5. Determination of Total Phenolic, Total Anthocyanin, and Vitamin C Contents

Total phenolic content was determined by using the Folin–Ciocalteu reagent, and the absorbance was measured at 765 nm [58]. Quantification was based on a standard curve for gallic acid. The results were expressed as mg g-1 fresh weight. The total anthocyanin content was estimated using the pH differential method [58]. The fruit extract was diluted with pH 1.0 (0.025 M potassium chloride) and pH 4.5 (0.4 M sodium acetate) buffers. The absorbance was measured at both 510 nm and 700 nm. The results were obtained from the formula mentioned in the previous report [58], expressed as mg g^−1^ fresh weight. According to the method described by Perin et al. [59], vitamin C (VC) content was determined using High Performance Liquid Chromatography (HPLC; D-2000, Hitachi, Tokyo, Japan). The mobile phase was 0.1% oxalic acid solution at a 1.0 mL min^−1^ flow rate, and the detection wavelength was 254 nm. An external standard curve of _L_-ascorbic acid was used to quantify VC content, expressed as mg g^–1^ fresh weight. There were three replicates with 10 fruits per replicate performed for the control and HNW treatments.

### 4.6. Assay of Antioxidant Enzyme Activity, ABTS·^+^ and DPPH· Scavenging Activity

The activities of superoxide dismutase (SOD), catalase (CAT), ascorbate peroxidase (APX), guaiacol peroxidase (POD), and glutathione reductase (GR) were measured according to the previous methods [14,60]. Protein content was determined using the method of Bradford [61]. The 2,2′-azino-bis(3-ethylbenzothiazoline-6-sulfonic acid) (ABTS·^+^) radical and 1,1-diphenyl-2-picryl-hydrazyl radical (DPPH·) scavenging assays were performed according to the previous methods [62]. There were three replicates with 10 fruits per replicate performed for the control and HNW treatments.

### 4.7. Protein Extraction and Proteomic Analysis

Six fruits were mixed and ground using liquid nitrogen. Four replicates for each treatment were performed for tandem mass tag (TMT)-labeled quantitative proteomic analysis. According to the previous method [63], the total protein was extracted, and the concentration was measured by the Bradford method. After digestion and estimation, the peptides were labeled based on the manufacturer’s instructions for the TMT labeling kit (Thermo Scientific, Waltham, MA, USA). The labeled peptide fragments were fractionated using high pH reverse-phase chromatography and combined into 15 fractions. The mass spectra analysis was carried out on a Q Exactive HF-X mass spectrometer coupled with an Easy-nLC 1200 system (Thermo Scientific, America). Peptides were separated through a C18 analytical column (75 μm × 25 cm × 2 μm, C18, 100 Å). The flow rate was 300 nL min^−1^. For data-dependent mode analysis, each scan cycle consisted of one full-scan mass spectrum (R = 60 K, AGC = 3 × 10^6^, max IT = 20 ms, scan range = 350–1800 *m/z*), followed by 20 MS/MS events (R = 45 K, AGC = 1 × 10^5^, max IT = 100 ms). HCD collision energy and isolation window for precursor selection were set to 32 eV and 1.2 Da, respectively. The former target ion exclusion was set to 35 s. The raw data were analyzed with MaxQuant v1.6.6. Protein identification was performed using *Vaccinium darrowii* genome assembly (GCA_020921065.1) as a reference [64]. Differentially expressed proteins (DEPs) were selected using *t*-test (*p* < 0.05, n = 4) and fold-change > 1.2 or <0.83.

### 4.8. Quantitative Real-Time PCR

Total RNA in fruit tissues was isolated using FastPure Plant Total RNA Isolation Kit (Polysaccharides & Polyphenolics-rich; Vazyme, Nanjing, China). The concentration and quality were estimated with a NanoDrop 2000 spectrophotometer (Thermo Fisher Scientific, Wilmington, DE, USA). The cDNAs were synthesized using HiScript III RT SuperMix with gDNA wiper (Vazyme, Nanjing, China). qPCR was performed with TransStart Top Green qPCR SuperMix (TransGen Biotech, Beijing, China) and a real-time PCR system (Mastercycler ep^®^ realplex; Eppendorf, Hamburg, Germany). Relative expression levels of genes were normalized to reference genes (*Actin* and *GAPDH*) and presented as values relative to the control group using the 2^−ΔΔCT^ method [65]. Primers were shown in Supplementary Table S6.

### 4.9. Statistical Analysis

Values were presented as mean ± standard deviation (SD) from three independent experiments (except for proteomic analysis). Results were analyzed by *t*-test using Origin 2022. Differences were considered significant at * *p* < 0.05, ** *p* < 0.01, and *** *p* < 0.001 (*t*-test). MetaboAnalyst 5.0 (https://www.metaboanalyst.ca, accessed on 23 July 2023) was used for partial least squares discriminant analysis (PLS-DA) of fruit quality traits. The raw data were normalized by internal standard area (sample median, data transformation by cube root, and data scaling by auto scaling). Weighted protein co-expression network analysis (WPCNA) was performed using the WGCNA v1.69 package in R (v3.6.1) to identify important proteins that correlated most with blueberry main fruit traits. A soft threshold value, power of 9, was selected to transform the adjacency matrix into a scale-free network. Modules whose eigenproteins were highly correlated were merged with a mergeCutHeight of 0.25 (minimum module size = 50). Module-trait associations were evaluated by calculating the Pearson correlations. The top 20 proteins in connectivity ranking (K value) were selected in a given module, and their network was visualized using R igraph v1.2.4.2.

## 5. Conclusions

**Figure 6:**
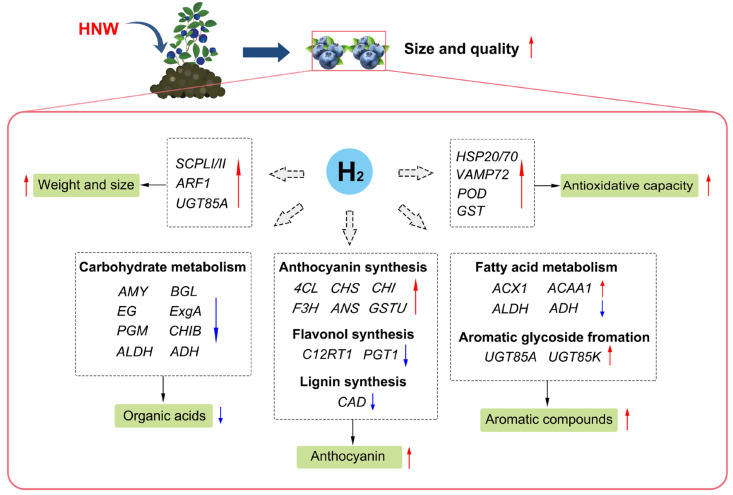
Schematic model describing hydrogen nanobubble water (HNW)-improved yield and quality of blueberries. 4CL: 4 coumarate CoA ligase; ACAA1: acetyl-CoA acyltransferase 1; ACX1: acyl-CoA oxidase; ADH: alcohol dehydrogenase; ALDH: alcohol dehydrogenase; AMY: α-amylase; ANS: anthocyanidin synthase; ARF1: ADP-ribosylation factor 1; BGL: β-glucosidase; CAD: cinnamyl alcohol dehydrogenase; CHI: chalcone isomerase; CHIB: endochitinase B; CHS: chalcone synthase; C12RT1: flavanone 7-O-glucoside 2′-O-β-L-rhamnosyltransferase; EG: endoglucanase; ExgA: glucan 1,3-β-glucosidase; F3H: flavanone 3-hydroxylase; GST: glutathione S-transferase; GSTU: tau class glutathione S-transferase; HSP20/70: heat shock 20/70kDa protein; PGM: phosphoglucomutase; PGT1: phlorizin synthase; POD: peroxidase; SCPLI/II: serine carboxypeptidase-like protein I/II; UGT85A/85K: UDP-glucosyltransferase 85A/85K; VAMP72: vesicle-associated membrane protein 72. The red and blue arrows indicate the upregulation and downregulation of gene exression, respectvely.

In summary, H_2_-base irrigation improves the fruit yield and quality of blueberries, including fruit weight and size, sugar–acid ratio, aroma compounds, anthocyanin content, and antioxidant capacity (Figure 6). Among these responses, SCPLI/II, UGT85A, HSP20/70, AMY, ADH, ACX1, and their encoding genes might be the candidate target proteins/genes. Further genetic work will focus on their detailed function identification. Overall, H_2_-based irrigation appears to be a promising approach for yield and quality improvement in horticultural products at a low carbon cost.

## Figures and Tables

**Table 1:** Effects of hydrogen nanobubble water (HNW) on soluble solids (SSC) and total soluble sugar (TSS) contents, titratable acidity (TA), SSC/TA, and TSS/TA in blueberry fruits.

Treatment	SSC (%)	TSS (%)	TA (%)	SSC/TA	TSS/TA
SW	11.57 ± 0.72	10.82 ± 0.71	0.48 ± 0.09	24.62 ± 2.95	23.06 ± 3.31
HNW	12.54 ± 0.21	11.2 ± 1.47	0.36 ± 0.02 *	34.57 ± 1.78 **	30.75 ± 3.14 *

Values are expressed as mean ± SD (three replicates and ten fruits per each). * *p* < 0.05 and ** *p* < 0.01 (*t*-test).

**Table 2:** Functional annotation of candidate proteins in different modules.

Module	ID	NCBI Accession No.	Regulated	Annotation
MEgreen	278	KAH7834522.1	up	Serine carboxypeptidase-like clade I
	1792	KAH7845398.1	up	Serine carboxypeptidase-like clade II
	1810	KAH7845536.1	up	UDP-glucosyltransferase 85A
	3005	KAH7854533.1	up	ADP-ribosylation factor 1
MEblue	2315	KAH7849624.1	down	α-Amylase
	4438	KAH7864589.1	down	Endoglucanase
	1216	KAH7840938.1	down	Endoglucanase
	694	KAH7837295.1	down	β-glucosidase
	1073	KAH7839787.1	down	Glucan endo-1,3-β-glucosidase1/2/3
	3786	KAH7860109.1	down	Glucan endo-1,3-β-glucosidase1/2/3
	2412	KAH7850303.1	down	Glucan 1,3-β-glucosidase
	303	KAH7834718.1	down	Phosphoglucomutase
	4573	KAH7865523.1	down	Aldehyde dehydrogenase (NAD+)
	4840	KAH7867194.1	down	Alcohol dehydrogenase (NADP+)
	101	KAH7833386.1	down	Alcohol dehydrogenase class-P
	4170	KAH7862891.1	down	Endochitinase B
	2116	KAH7848277.1	down	Endochitinase B
	4333	KAH7863910.1	down	Chitinase
MEturquoise	543	KAH7836374.1	up	Acetyl-CoA acyltransferase 1
	3870	KAH7860773.1	up	Acyl-CoA oxidase
	3896	KAH7860965.1	up	Cyanohydrin UDP-glucosyltransferase
	719	KAH7837441.1	up	HSP20 family protein
	3481	KAH7857874.1	up	Heat shock 70kDa protein
	4457	KAH7864708.1	up	Peroxidase
	3694	KAH7859366.1	up	Glutathione S-transferase
MEsteelblue	39	KAH7832952.1	up	HSP20 family protein
	4124	KAH7862607.1	up	Vesicle-associated membrane protein 72

## Data Availability

The data that support the findings of this study are available in the supplementary files. The proteomics data in this study are available in FigShare (DOI: 10.6084/m9.figshare.27216609).

## Supplementary Materials

The following supporting information can be downloaded at https://www.mdpi.com/article/10.3390/plants14142137/s1, Figure S1: KEGG enrichment analysis of differentially expressed proteins of hydrogen nanobubble water (HNW)-irrigated blueberry fruits (*p*-value ranks of the 20 top); Figure S2: Co-expressed protein clustering and module construction; Table S1: Summary of protein identification; Table S2: Summary of differentially expressed proteins; Table S3: Differentially expressed proteins in blueberry fruits irrigated with/without HNW; Table S4: Timing and quantity of HNW irrigation; Table S5: Nutrient solution composition; Table S6: Primers for qPCR.
