# Supplementary material for: Molecular Hydrogen Improves Blueberry Main Fruit Traits via Metabolic Reprogramming
Source: Plants (Basel). 2025 Jul 10;14(14):2137. doi: 10.3390/plants14142137 (PMC12298964; doi:10.3390/plants14142137)
Supplement: Supplementary file 1 [file plants-14-02137-s001.zip › plants-3705872-supplementary.pdf]

Supplementary Information

Title:

Molecular hydrogen improves blueberry main fruit traits via metabolic reprogramming

Longna Li[1], Jiaxin Gong[1], Ke Jiang[1], Liqin Huang[2], Lijun Gan[1], Yan Zeng[3], Xu Cheng[3], Didier Pathier[3], Wenbiao Shen[1, *]

[1] *College of Life Sciences, Laboratory Center of Life Sciences, Nanjing Agricultural University, Nanjing 210095, China*

[2] *College of Sciences, Nanjing Agricultural University, Nanjing 210095, China*

[3] *Life Science Group, Air Liquide (China) R&D Co., Ltd, Shanghai 201108, China*

[*]**Corresponding author at:** College of Life Sciences, Laboratory Center of Life Sciences, Nanjing Agricultural University, Nanjing 210095, China

E-mail address: wbshenh@njau.edu.cn (W. Shen)

Table S1 Summary of protein identification

Total spectra	Spectra (PSM)	Peptides	Unique peptides	Proteins
309,727	52,935	22,548	18,000	4,756

Table S2 Summary of differentially expressed proteins

Compared name	Up regulated	Down regulated	Total DEPs
Con vs HNW	59	83	142

Table S3 Differentially expressed proteins in blueberry fruits irrigated with/without HNW

ID	NCBI Accession No.	Regulated	K number	KEGG pathways
29	KAH7832887.1	down	K24028	--
39	KAH7832952.1	up	K13993	ko04141
91	KAH7833276.1	down	--	--
101	KAH7833386.1	down	K18857	ko00010;ko00071;ko00350;ko00592;ko00620;ko01100;ko01110
180	KAH7833859.1	down	--	--
211	KAH7834055.1	up	--	--
235	KAH7834205.1	down	K00799	ko00480;ko01100
278	KAH7834522.1	up	K16296	--
303	KAH7834718.1	down	K01835	ko00010;ko00030;ko00052;ko00230;ko00500;ko00520;ko01100;ko01110;ko01250
343	KAH7834990.1	down	--	--
372	KAH7835201.1	down	K13473	ko04626
389	KAH7835320.1	down	K05894	ko00592;ko01100;ko01110
410	KAH7835466.1	up	K20293	--
444	KAH7835702.1	down	K03327	--
486	KAH7835933.1	up	K23341	--
524	KAH7836220.1	down	--	--
543	KAH7836374.1	up	K07513	ko00071;ko00280;ko00592;ko01040;ko01100;ko01110;ko01212;ko04146
552	KAH7836418.1	down	K10133	--
561	KAH7836473.1	up	K00679	ko00561;ko01100
691	KAH7837287.1	up	K10398	--
694	KAH7837295.1	down	K05349	ko00460;ko00500;ko00999;ko01100;ko01110
703	KAH7837350.1	down	K22395	ko00940;ko01100;ko01110
719	KAH7837441.1	up	K13993	ko04141
910	KAH7838758.1	down	K20628	--
928	KAH7838834.1	down	K22395	ko00940ko01100;ko01110
932	KAH7838862.1	up	K17778	--
956	KAH7839017.1	up	K14709	--
972	KAH7839155.1	up	K10704	--
1002	KAH7840572.1	down	K20628	--
1033	KAH7839546.1	up	K05928	ko00130;ko01100;ko01110;ko01240
1043	KAH7839624.1	down	K13080	ko00941;ko00944
1073	KAH7839787.1	down	K19891	ko00500;ko01100
1089	KAH7839871.1	up	--	--

ID	NCBI Accession No.	Regulated	K number	KEGG pathways
1106	KAH7840027.1	down	K24184	--
1135	KAH7840273.1	down	K19747	--
1174	KAH7840566.1	down	--	--
1190	KAH7840727.1	up	K01859	ko00941;ko01100;ko01110
1199	KAH7840789.1	up	K22683	--
1216	KAH7840938.1	down	K01179	ko00500;ko0110
1256	KAH7841239.1	up	K16860	ko00564;ko00565;ko01100;ko01110
1415	KAH7842409.1	down	K05387	--
1417	KAH7842417.1	up	K12489	ko04144
1447	KAH7842724.1	up	K07305	--
1451	KAH7842734.1	up	--	--
1456	KAH7842777.1	down	K16040	ko00945;ko01110
1584	KAH7843739.1	down	K05387	--
1594	KAH7844738.1	down	K05387	--
1595	KAH7843807.1	down	K05387	--
1602	KAH7843868.1	up	K01081	ko00230;ko00240;ko00760;ko01100;ko01110;ko01232
1627	KAH7844107.1	down	K22588	ko00380;ko01100;ko01110
1660	KAH7844398.1	up	--	--
1745	KAH7844960.1	down	K05387	--
1792	KAH7845398.1	up	K16297	--
1795	KAH7845436.1	down	K13963	--
1810	KAH7845536.1	up	K23452	ko00908;ko01110
1821	KAH7845617.1	down	K01455	ko00460;ko00630;ko00910;ko0110;ko01200
1833	KAH7845687.1	up	K00799	ko00480;ko01100
1864	KAH7847338.1	down	K11450	--
1903	KAH7846388.1	down	K13513	ko00561;ko00564;ko01100;ko01110
1956	KAH7846817.1	down	K00413	ko00190;ko01100
2018	KAH7847353.1	up	K00679	ko00561;ko01100
2030	KAH7847441.1	up	K24917	--
2035	KAH7847458.1	down	K00472	ko00330;ko01100
2075	KAH7847879.1	down	K22683	--
2081	KAH7847925.1	down	K23452	ko00908;ko01110
2116	KAH7848277.1	down	K20547	ko00520;ko01100;ko04016
2172	KAH7848650.1	down	K03097	ko03008;ko04712
2201	KAH7848892.1	down	K15333	--
2276	KAH7849385.1	down	--	--
2282	KAH7849437.1	up	K01883	ko00970

ID	NCBI Accession No.	Regulated	K number	KEGG pathways
2304	KAH7849559.1	up	K00121	ko00010;ko00071;ko00350;ko00620;ko01100;ko01110;ko01200
2315	KAH7849624.1	down	K01176	ko00500;ko01100;ko0111
2412	KAH7850303.1	down	K01210	ko00500;ko01100
2433	KAH7850431.1	down	K20667	--
2460	KAH7850644.1	down	K13091	--
2486	KAH7850859.1	down	K00924	--
2544	KAH7851302.1	down	K01757	ko00901;ko01100;ko01110
2623	KAH7851800.1	up	K03671	--
2627	KAH7851828.1	up	K08967	ko00270;ko01100
2709	KAH7852441.1	up	K20728	ko04016
2716	KAH7852499.1	down	--	--
2803	KAH7853087.1	up	K21804	--
2883	KAH7853693.1	down	K05019	--
2887	KAH7853711.1	up	K02910	ko03010
3005	KAH7854533.1	up	K07937	ko04144
3009	KAH7855349.1	down	K22845	ko00941;ko01110
3124	KAH7855328.1	up	K13412	ko04626
3138	KAH7855424.1	up	--	--
3227	KAH7856093.1	down	K08869	--
3287	KAH7861523.1	down	K23806	--
3320	KAH7856718.1	down	K25108	--
3357	KAH7856972.1	up	K00547	ko00270;ko0110;ko01110
3379	KAH7857127.1	down	K13126	ko03015;ko03018
3422	KAH7857476.1	down	K17302	--
3481	KAH7857874.1	up	K09489	--
3484	KAH7857931.1	down	K22683	--
3522	KAH7858142.1	up	K12858	ko03040
3547	KAH7858956.1	down	K00276	ko00260;ko00350;ko00360;ko00410;ko00950;ko00960;ko01100;ko01110
3694	KAH7859366.1	up	K00799	ko00480;ko01100
3730	KAH7859652.1	down	K18195	--
3766	KAH7861265.1	up	K14771	--
3786	KAH7860109.1	down	K19891	ko00500;ko01100
3870	KAH7860773.1	up	K00232	ko0007;ko00410;ko00592;ko00640;ko01040;ko01100;ko01110;ko01200;ko01212;ko04146
3893	KAH7860943.1	down	--	--

ID	NCBI Accession No.	Regulated	K number	KEGG pathways
3896	KAH7860965.1	up	K16266	ko00460;ko01100;ko01110
3911	KAH7861090.1	down	K03098	--
3923	KAH7861188.1	down	K25075	ko00943;ko01100;ko01110
3944	KAH7861317.1	up	K00901	ko00561;ko00564;ko01100;ko01110;ko04070
3968	KAH7861455.1	down	--	--
4041	KAH7862074.1	up	K01057	ko00030;ko01100;ko01110;ko01200
4046	KAH7862106.1	down	K00855	ko00710;ko01100;ko01200
4092	KAH7862407.1	down	K12462	--
4105	KAH7862490.1	up	K08678	ko00520;ko01100;ko01250
4113	KAH7862538.1	down	K10779	--
4124	KAH7862607.1	up	K08511	--
4153	KAH7862788.1	up	--	--
4170	KAH7862891.1	down	K20547	ko00520;ko01100;ko04016
4175	KAH7862928.1	up	K22683	--
4297	KAH7863710.1	down	K03141	ko03022;ko03420
4316	KAH7863816.1	down	K10576	ko04120
4326	KAH7863867.1	up	K05291	ko00563;ko01100
4333	KAH7863910.1	down	K01183	ko00520;ko01100
4360	KAH7864058.1	down	K13466	ko04626
4438	KAH7864589.1	down	K01179	ko00500;ko01100
4445	KAH7864616.1	down	--	--
4457	KAH7864708.1	up	K00430	ko00940;ko01100;ko01110
4462	KAH7864733.1	down	K23050	--
4491	KAH7865016.1	down	K11968	--
4534	KAH7865311.1	down	K01673	ko00910;ko01100
4542	KAH7865351.1	down	K00030	ko00020;ko01100;ko01110;ko01200;ko01210;ko01230
4573	KAH7865523.1	down	K00128	ko00010;ko00053;ko00071;ko00280;ko00310;ko00330;ko00340;ko00380;ko00410;ko00561;ko00620;ko00770;ko00903;ko01100;ko01110;ko01240
4576	KAH7865536.1	down	K12349	ko00600;ko01100
4636	KAH7865851.1	down	K08245	--
4667	KAH7865999.1	down	K01728	ko00040;ko01100
4687	KAH7866158.1	up	K22418	--
4708	KAH7866321.1	up	K15223	--
4721	KAH7866415.1	up	K13993	ko04141
4722	KAH7866420.1	up	K16280	--
4743	KAH7866600.1	up	K14497	ko04016;ko04075

ID	NCBI Accession No.	Regulated	K number	KEGG pathways
4808	KAH7866979.1	up	K10839	ko03420;ko04141
4830	KAH7867122.1	up	K00276	ko00260;ko00350;ko00360;ko00410;ko00950;ko00960;ko01100;ko01110
4840	KAH7867194.1	down	K00002	ko00010;ko00040;ko00053;ko00561;ko00620;ko01100;ko01110;ko01240

Table S4 Timing and quantity of HNW irrigation

Irrigation time	Consumption (t/d)
28/02/2022-14/3/2022	0.48
15/3/2022-4/5/2022	0.96
5/5/2022-16/6/2022	1.44

Table S5 Nutrient solution composition

Ingredients	Dosage (mg/L)
NH_4^+	190~3580
NO_3^-	1795~7750
PO_4^{3-}	500~1035
K^+	1590~2000
Ca^{2+}	36~1030
SO_4^{2-}	20~830
Fe^{3+}	1.2~200
Mg^{2+}	25~270
Mn^{2+}	1.3~30
Zn^{2+}	0.3~36
Cu^{2+}	0.02~10
MoO_4^{2-}	0.03~15

Table S6 Primers for qPCR

Gene (NCBI accession No.)		Primer sequence (5'→3')
VmGAPDH (AY123769.1)	F	GGTTATCAATGATAGGTTTGGCA
	R	CAGTCCTTGCTTGATGGACC
VcACT (AB694898.1)	F	GCTTTGGTGGTGAGTTTCTGT
	R	CATTCATAAGCCCCAACTCAGC
VcPAL (MH321457.1)	F	AACTCCGACAAGTCCTGGTG
	R	CGACTTCCTTGGGCAAAACG
VcC4H (MH321458.1)	F	CGCCAATAACCCCGAGACTT
	R	GGTACCTGAAGTCGTTCCCG
Vc4CL (MH321459.1)	F	AATGCTATGTGGGCTCCGTG
	R	ACCACCGGACTTTTGGCTAT
VaCHS (AB694903.1)	F	CCGACTACCAGCTCACCAAG
	R	AGCCCCCTTGTTGTTCTCTG
VcCHI (MH321461.1)	F	GGGGTGTGTCTCCTTCAACC
	R	TCAAAACGGCGCAAACTCAG
VaF3H (AB610765.1)	F	ATACCCACTTCGCAACCGAG
	R	CAACACGTCGAGCATCTTGC
VaF3'H (AB694901.1)	F	TTGAAGACGCACGACCAGAA
	R	TCTTCCTAAGCATCCGCCAC
VaF3'5'H (MH321464.1)	F	GTCGGTTGCATGGATGGACT
	R	AGCCGACTCACTATGTCCCT
VaDFR (AB610763.1)	F	AGATGCTAACAGTGGGCTGG
	R	CTCGAACAACATAGCCCCGT
VcANS (JN654701.1)	F	ATGCGAATCACCTGAGAGCC
	R	TGACGAGTTCGTCCTTTCCG
VaUFGT (AB694900.1)	F	GCTCTCTGTCGGCTCATTGT
	R	TTCCATACACGACTCCGCTG
VcGSTU (KT601064.1)	F	TGATGACGTTTGGGCCTCTG
	R	GCCTTCTTGGCTTCCTCTGT
VdSCPLI (KAH7834522.1)	F	TTCGGAAATTCTGGTGCCGA
	R	GCATCTCGCCCATCTGTCTT
VdSCPLII (KAH7845398.1)	F	TGATGGTGCTCCTCTGGGTA
	R	GCAATTCATCGGTGGCATCC
VdARF1 (KAH7854533.1)	F	CGTTGACGCTTTCTCTCCAC
	R	CCAAGCATCACAACCCTCATC
VdAMY (KAH7849624.1)	F	TGGATGAACTGGTTAAGGTCC
	R	CCCATCCGATTCGTACGCTA
VdEG (KAH7840938.1)	F	ACCGCCATTCCTCCTCATTC
	R	GGATCGCTGGCCTTCAAAGA
VdExgA (KAH7850303.1)	F	GGCACCCTATTGGAAAACAAAAC
	R	GAGTGACCCTACCGAAGGC
VdBGL (KAH7837295.1)	F	GGCGAAGTGGAACGAGGTAT
	R	TCACGGTCATTCGAGACAGC

Gene (NCBI accession No.)		Primer sequence (5’→3’)
VdPGM (KAH7834718.1)	F	ACACTCCTCGGAACATCACAG
	R	AGCGCATTGAATGTTGCCTG
VdCHIB (KAH7862891.1)	F	AAAGCGCTTCCAATGTTCGT
	R	TTTCCGCCATTCAAGAGGCT
VdALDH (KAH7865523.1)	F	GACTGCTGAGCAAACCCCAT
	R	GCACAACCTTACCTGTGGCA
VdADH (KAH7867194.1)	F	ATGGAGAGCAATGGAGGAGC
	R	AGCTTTACAGAACGCACGAA
VdACX1 (KAH7860773.1)	F	CACCCTAGGGGAGCTTGTTG
	R	GACTTCCGGCTGATTTGCAG
VdACAA1 (KAH7836374.1)	F	GGACCAGGCTGCTGTTGTAT
	R	TCACAACCTGCTTCTCCACG
VdUGT85A (KAH7845536.1)	F	CTCGCCAACAGCAAGCAAAA
	R	CAACAGAAGGGTGGCAGAGA
VdUGT85K (KAH7860965.1)	F	ACGTGACCGACCCTCAAATC
	R	TCTCAAGTGGTCCCCACTCA
VdHSP20 (KAH7832952.1)	F	TCGGGTACTCCTTCACGTCC
	R	CTCCTGGACCCCAACAATGC
VdHSP70 (KAH7857874.1)	F	CGGAGATGAATCCGTGGGAG
	R	CCAGGCACCAAGACCCTAAG
VdVAMP72 (KAH7862607.1)	F	GGACCCAGGGTACCCAAATG
	R	TCTACAAGCAATTTGCCGCC

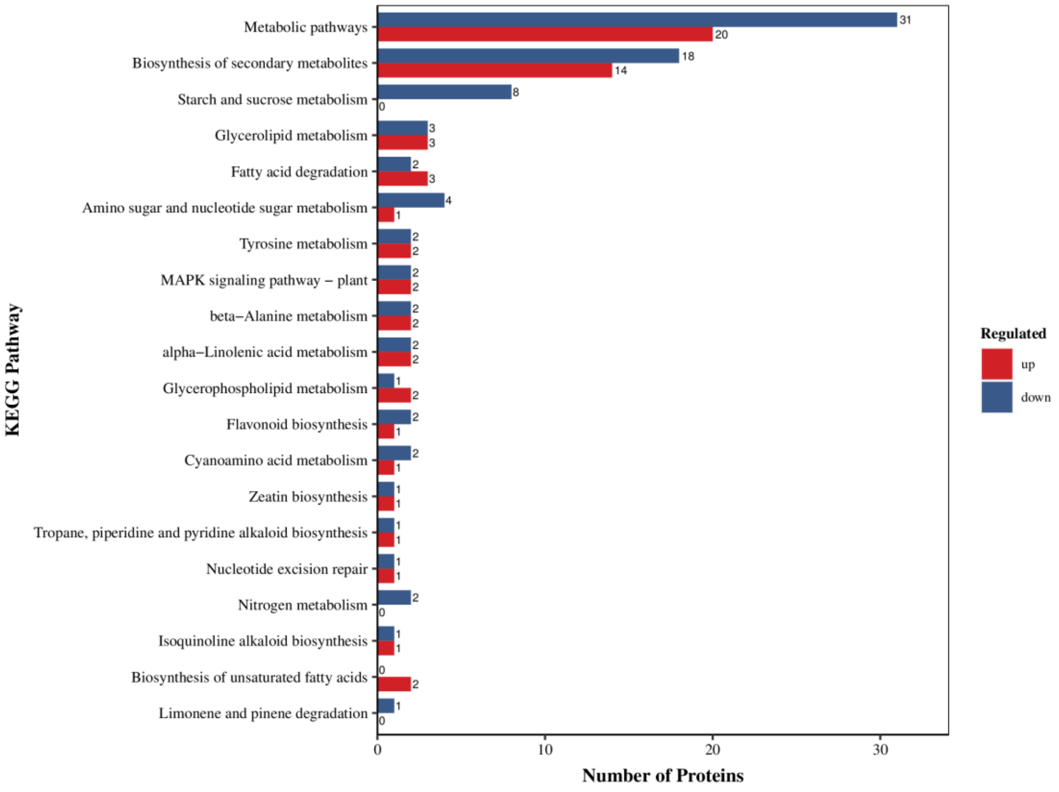

Figure S1. KEGG enrichment analysis of differentially expressed proteins of hydrogen nanobubble water (HNW)-irrigated blueberry fruits (*p*-value ranks of the 20 top).

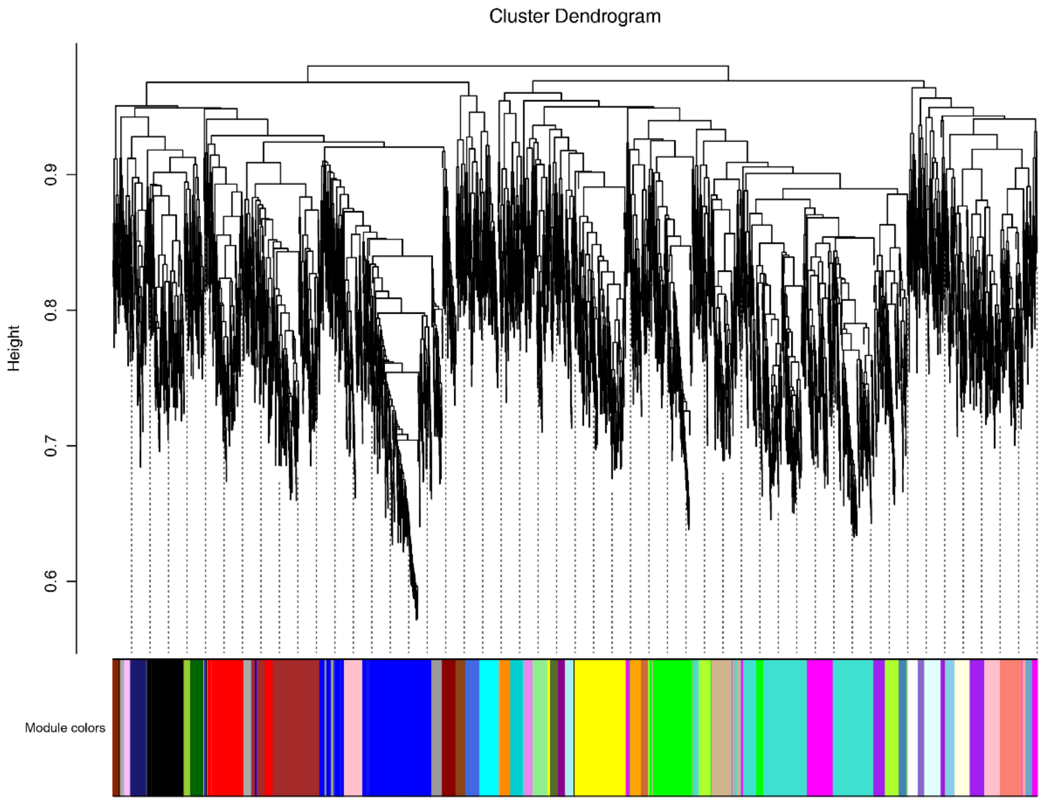

Figure S2. Co-expressed protein clustering and module construction. Each leaf of tree corresponds to one protein. The major tree branches constitute 41 modules, labeled with different colors.
